# Enteroendocrine Hormone Secretion and Metabolic Control: Importance of the Region of the Gut Stimulation

**DOI:** 10.3390/pharmaceutics12090790

**Published:** 2020-08-21

**Authors:** Cong Xie, Karen L. Jones, Christopher K. Rayner, Tongzhi Wu

**Affiliations:** 1 Adelaide Medical School and Centre of Research Excellence (CRE) in Translating Nutritional Science to Good Health, The University of Adelaide, Adelaide 5005, Australia; c.xie@adelaide.edu.au (C.X.); karen.jones@adelaide.edu.au (K.L.J.); chris.rayner@adelaide.edu.au (C.K.R.); 2 Endocrine and Metabolic Unit, Royal Adelaide Hospital, Adelaide 5005, Australia; 3 Department of Gastroenterology and Hepatology, Royal Adelaide Hospital, Adelaide 5005, Australia; 4 Institute of Diabetes, School of Medicine, Southeast University, Nanjing 210009, China

**Keywords:** nutrient digestion, nutrient absorption, gastrointestinal hormone, postprandial glycaemia, energy intake, region of the gut, obesity, type 2 diabetes

## Abstract

It is now widely appreciated that gastrointestinal function is central to the regulation of metabolic homeostasis. Following meal ingestion, the delivery of nutrients from the stomach into the small intestine (i.e., gastric emptying) is tightly controlled to optimise their subsequent digestion and absorption. The complex interaction of intraluminal nutrients (and other bioactive compounds, such as bile acids) with the small and large intestine induces the release of an array of gastrointestinal hormones from specialised enteroendocrine cells (EECs) distributed in various regions of the gut, which in turn to regulate gastric emptying, appetite and postprandial glucose metabolism. Stimulation of gastrointestinal hormone secretion, therefore, represents a promising strategy for the management of metabolic disorders, particularly obesity and type 2 diabetes mellitus (T2DM). That EECs are distributed distinctively between the proximal and distal gut suggests that the region of the gut exposed to intraluminal stimuli is of major relevance to the secretion profile of gastrointestinal hormones and associated metabolic responses. This review discusses the process of intestinal digestion and absorption and their impacts on the release of gastrointestinal hormones and the regulation of postprandial metabolism, with an emphasis on the differences between the proximal and distal gut, and implications for the management of obesity and T2DM.

## 1. Introduction

As the key interface between ingested nutrients and the body, the gastrointestinal tract is now recognised to play a central role in regulating postprandial metabolism. During the fasting state, ghrelin is released from the gastric Gr-cells to drive food intake [1]. After meal ingestion, the stomach accommodates the nutrients, grinds digestible solids into small particles, and releases the resultant chyme into the small intestine in a regulated fashion to optimise intestinal digestion and absorption. It is now widely appreciated that distinctive enteroendocrine cells (EECs) scattered along the gastrointestinal tract, comprising up to 1% of the gut epithelium, constitute the largest endocrine organ in the body, accounting for the release of an array of peptides that orchestrate appetite, energy intake and the blood glucose responses to meals [2]. Of particular importance are cholecystokinin (CCK) and glucose-dependent insulinotropic polypeptide (GIP) released from the upper small intestine, and glucagon-like peptide-1 (GLP-1) and peptide YY (PYY) secreted mainly from the distal gut. These integrated hormonal responses convey important regulatory signals governing subsequent gastric emptying, insulin and glucagon secretion from the pancreas, energy intake, and postprandial glycaemic control. Stimulation of gastrointestinal postprandial hormone secretion, therefore, represents an attractive strategy for the management of metabolic disorders, such as obesity and type 2 diabetes mellitus (T2DM) [2]. Given that the distribution of the respective types of EECs varies substantially along the gastrointestinal tract, the region of the gut exposed to intraluminal stimuli is likely to be of major relevance to the secretion profile of gastrointestinal hormones and associated metabolic responses. This review discusses nutrient digestion and absorption along the gastrointestinal tract and how these processes influence the secretion of GIP, CCK, GLP-1 and PYY, and highlights the importance of which region of the gut is stimulated to the secretory profiles of these gastrointestinal hormones, the regulation of postprandial metabolism, and the implications for the management of obesity and T2DM. Other hormones, such as ghrelin and leptin, are also important metabolic regulators, but are not specifically discussed as they are outside the scope of this review.

## 2. Nutrient Transport, Digestion, and Absorption

Following meal ingestion, the stomach stores the ingested content and grinds digestible solids into small particles prior to delivering them into the small intestine. The latter occurs at a relatively constant caloric rate (in the range of 1–4 kcal/min in healthy individuals), driven by antral and duodenal contractions against tonic and phasic pyloric pressures [3], to optimise the subsequent digestion and absorption of nutrients in the small intestine. Due to the inactivation of salivary amylase in the gastric environment, there is limited digestion of carbohydrate in the stomach, whereas fat and protein are digested into lipid emulsions [4] and oligopeptides [5], respectively. Upon entering the duodenum, nutrients stimulate the release of a range of digestive enzymes from the exocrine pancreas and bile acids from the gallbladder, influenced by both the load and composition of the meal. Starch is broken down by pancreatic α-amylase and disaccharidases into glucose and other monosaccharides (e.g., fructose and galactose) [6]. Dietary fat (90–95% in the form of triglycerides) is digested by pancreatic lipase, a process relying largely on emulsification by bile acids, to form monoacylglycerol, glycerol and free fatty acids [4]. Digestion of protein involves both pancreatic enzymes, including chymotrypsin and trypsin, and aminopeptidases secreted by the small intestine mucosa, and yields individual amino acids, dipeptides and tripeptides [5].

The digestive products are transported by peristalsis and absorbed by passive diffusion and/or active transport via distinctive transporters at specific regions of the gut. For example, absorption of glucose involves both active transport from the lumen into the enterocytes via sodium-glucose cotransporter 1 (SGLT-1) and facilitated diffusion across the basolateral side of enterocytes through the glucose transporter 2 (GLUT2), taking place predominantly in the upper small intestine [7,8,9,10]. Unlike glucose, fructose is absorbed mainly through GLUT5 [11], which is well expressed in both the human jejunum and ileum [12]. Dietary fat typically binds to bile acids to form mixed micelles, which are absorbed by fatty acid transport proteins (FATPs) (e.g., FATP4 and FAT/CD36) and Niemann-Pick C1 like-1 (NPC1L1) [13,14]. Although a small fraction of bile acids are absorbed passively in the jejunum, the majority of them (~90%) are actively absorbed in the ileum through the apical sodium-dependent bile acid transporter (ASBT) [15]. The uptake of amino acids depends on a variety of “amino acid transport systems” that preferentially transport amino acids of similar biophysical properties [16,17], whereas dipeptides and tripeptides are absorbed via the proton-dependent intestinal peptide transporter 1 (PEPT1) [18]. The large intestine hosts a diversity of gut bacteria, which are involved in the fermentation of products that escape digestion/absorption in the small intestine, such as dietary fibre, resistant starches and proteins, leading to the production of short-chain fatty acids (SCFAs) which can be absorbed through facilitated diffusion [19,20,21].

## 3. Secretion and Actions of Gastrointestinal Hormones

**Figure 1:**
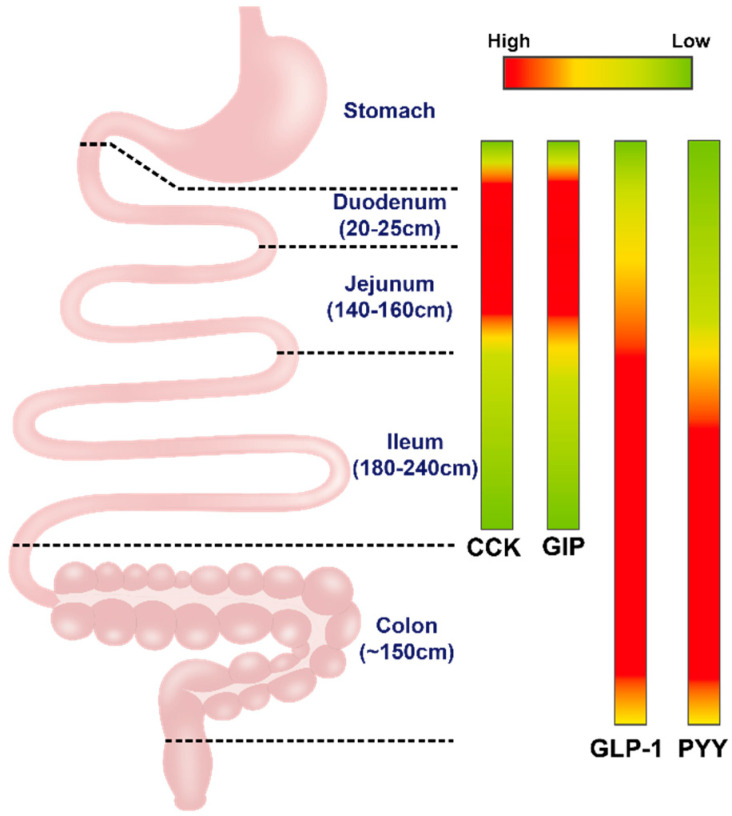
The density of enteroendocrine cells (EECs) secreting cholecystokinin (CCK), glucose-dependent insulinotropic polypeptide (GIP), glucagon-like peptide 1 (GLP-1) and peptide YY (PYY) in the duodenum, jejunum, ileum and colon.

**Figure 2:**
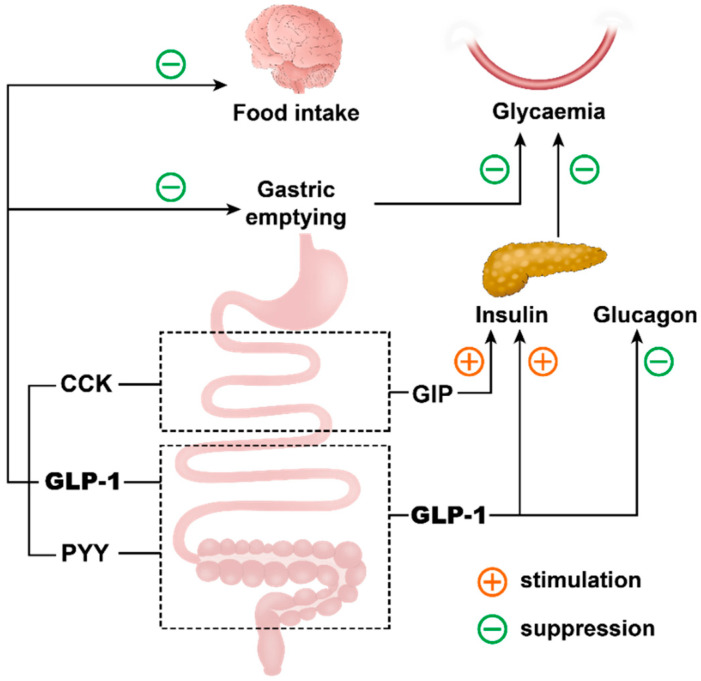
The roles of gastrointestinal hormones, including cholecystokinin (CCK), glucose-dependent insulinotropic polypeptide (GIP), glucagon-like peptide 1 (GLP-1) and peptide YY (PYY), released in response to meal ingestion, in the regulation of gastric emptying, postprandial glycemia and energy intake.

While intestinal EECs maintain a low secretory profile during fasting, the interaction between intraluminal contents and EECs during the digestive process represents the main driver of the secretion of gastrointestinal hormones. The latter, including GLP-1, GIP, CCK and PYY released from distinctive EECs in various regions of the gastrointestinal tract (Figure 1), are now recognised as key regulators of energy intake and postprandial glucose metabolism (Figure 2).

EECs are equipped with a variety of chemo-sensors linking the sensing of intraluminal stimuli to the secretion of gastrointestinal hormones. For example, carbohydrates can be detected by both their sweet taste through sweet taste receptors (STRs) [22,23,24], and via the glucose transporters, SGLT-1 and GLUT2 [25,26], although stimulation of STRs by artificial sweeteners alone does not seem to be sufficient to induce GLP-1 or GIP secretion in humans [23,27]. EEC sensing of intraluminal fat is dependent on the degree of its digestion [28,29,30] and involves a number of G protein-coupled receptors (GPRs), such as GPR40, GPR119 and GPR120 [31,32,33], and intestinal fat transporters FATP4 and FAT/CD36 [34,35]. Amino acids are detected by the calcium-sensing receptors (CasR) [25,36] and amino acid transporters [37,38]. Non-nutritive compounds are also effectively sensed by EECs. In particular, bile acids are known to induce GLP-1 and PYY secretion via inhibition of nuclear farnesoid X receptor (FXR) [39] and/or stimulation of Takeda G-protein coupled receptor 5 (TGR-5) [40,41]. Of note, TGR5 is expressed on the basolateral membrane of EECs [42], such that intestinal bile acid absorption is necessary to achieve TGR5 activation [41,42]. There is recent evidence that a group of specialized GPRs responsible for the sensing of bitter taste (i.e., bitter taste receptors; BTRs) are also abundantly expressed on EECs. Activation of BTRs on EECs by a variety of natural or chemosynthetic bitter taste compounds, therefore, has the potential to trigger the secretion of gastrointestinal hormones [43].

### 3.1. Glucagon-Like Peptide-1 (GLP-1)

GLP-1 is secreted from the enteroendocrine L-cells located mainly in the ileum and colon in response to each of the macronutrients, although fat, relative to glucose and protein, is generally more potent at stimulating GLP-1 secretion when administered into the duodenum in humans [44,45]. However, in patients who have undergone Roux-en-Y gastric bypass, oral ingestion of glucose was shown to be more effective than fat or protein to stimulate GLP-1 secretion [46]. The discrepancy observed in the latter is likely to be attributable to the influence of gastric emptying and digestion of fat or protein. Other bioactive compounds released into the lumen following meal ingestion, such as bile acids, are also responsible for postprandial GLP-1 secretion [41,47]. After its secretion, GLP-1 is rapidly inactivated by the enzyme dipeptidyl peptidase IV (DPP-4) with a half-life of 1–2 min, such that only 10–15% intact GLP-1 reaches the peripheral circulation [48,49]. While obesity is associated with attenuated GLP-1 secretion, accumulating evidence suggests that the latter is otherwise unaltered in patients with T2DM [50,51]. Importantly, the action of GLP-1 is also relatively well preserved in T2DM [51].

GLP-1 binds to its receptor, expressed in a variety of metabolic tissues, to regulate glucose, lipid and energy metabolism. Within the pancreas, GLP-1 stimulates insulin secretion from the pancreatic β-cells and suppresses glucagon secretion from the α-cells in a glucose-dependent manner [52]. For this reason, GLP-1-based glucose-lowering therapies, in general, entail a low risk of hypoglycaemia. Although there is preclinical evidence that GLP-1 receptor signalling is involved in β-cell survival and regeneration [53], such effects have not been established in humans. Within the liver, GLP-1-signalling is linked to the control of endogenous glucose production, an effect that can be independent of changes in insulin or glucagon [54]. Moreover, GLP-1 slows gastric emptying in both healthy individuals and those with T2DM [55,56,57,58]. That the reduction in postprandial glycaemia induced by exogenous GLP-1 is associated with less, rather than more, postprandial insulin secretion suggests that the slowing of gastric emptying outweighs its insulinotropic effect in controlling postprandial glycaemia [59]. In contrast to the GLP-1 receptor agonists, the DPP-4 inhibitors—which prolong the half-life of endogenous GLP-1—have little, if any, effect on gastric emptying [60,61].

Effects of GLP-1 signalling on lipid metabolism have been noted in both preclinical and clinical studies. GLP-1 has been shown to inhibit the production of lipid proteins (e.g., apolipoprotein B-48 (apob-48)) that are involved in the synthesis and transport of chylomicrons in the enterocytes, thereby improving lipid metabolism in rodents [62,63]. Similarly, the GLP-1 receptor agonist, exenatide, and the DPP-4 inhibitor, sitagliptin, have been shown to reduce plasma apob-48 concentrations in humans [64,65], while in obesity, treatment with GLP-1 receptor agonists improves dyslipidaemia [66].

GLP-1 also has the capacity to regulate energy intake and expenditure. Both exogenous GLP-1 and the GLP-1 receptor agonists suppress energy intake [50,67], and this effect has been shown to be mediated primarily via vagal afferents [68,69] and the activation of GLP-1 receptors in the central nervous system [70,71]. GLP-1 receptor agonists are therefore often associated with weight loss in both obesity and T2DM [48,72]. The role of GLP-1 in the regulation of energy expenditure is controversial. In mice, GLP-1 receptor agonists have been reported to induce browning of white adipose tissue and increase β-oxidation of fatty acids [73,74,75], and administration of both GLP-1 receptor agonists and DPP-4 inhibitors increases energy expenditure and reduces body weight [75,76,77,78]. However, a recent meta-analysis suggests that GLP-1 receptor agonists have little, if any, effect on energy expenditure in humans [79]. Although inhibition of DPP-4 by vildagliptin was found to augment the energy expenditure response to an intraduodenal fat infusion in healthy humans [80], this effect was not evident in patients with T2DM [81]. That antagonism of GLP-1 signalling by exendin (9–39) increased energy expenditure in the latter group during treatment with vildagliptin suggests that endogenous GLP-1 signalling may rather be associated with suppression of energy expenditure [81].

### 3.2. Glucose-Dependent Insulinotropic Polypeptide (GIP)

GIP is released from the enteroendocrine K-cells, distributed predominantly in the upper small intestine [51]. GIP is also co-expressed with GLP-1 in a subset of “K/L-cells” in the duodenum and proximal jejunum [82,83]. Similar to GLP-1, GIP is released in response to macronutrients—with fat being a more potent stimulus than glucose or protein [44]—and rapidly inactivated by DPP-4 after secretion [51,84]. However, the secretion of GIP does not seem to be affected by T2DM or obesity [50].

In health, GIP stimulates insulin secretion in a glucose-dependent manner by binding to the GIP receptor expressed on the pancreatic β-cells [85], which contributes equally with GLP-1 to the augmented insulin response that is observed during enteral glucose administration when compared to an “isoglycaemic” intravenous glucose infusion, i.e., the so-called the “incretin effect”. However, the insulinotropic effect of GIP is markedly diminished in patients with T2DM [49,86]. Unlike GLP-1, GIP stimulates glucagon secretion from the pancreatic α-cells, particularly in the face of hypoglycaemia [87], and has little effect on appetite [88] or gastrointestinal motility [89]. Moreover, GIP exhibits numerous extra-glycaemic actions; blockade of GIP signalling in mice preferentially increases fat oxidation [90,91,92], reduces fat accumulation in adipocytes [90,92,93] and skeletal muscle [91,92], decreases triglyceride deposition in the liver [90,93], and prevents the development of obesity [90,91,94] in the context of overfeeding. In line with these findings, the antagonism of the GIP receptor is associated with reduced blood flow and triglyceride deposition in adipose tissue in healthy subjects [95]. Although compounds that display dual GIP and GLP-1 receptor agonism appear to be more effective for weight loss and glycaemic control than the GLP-1 receptor agonists, liraglutide and dulaglutide, in patients with T2DM [96,97], the relative contribution of GIP signalling to this superiority remains to be characterized in humans. Counterintuitively, acute administration of exogenous GIP failed to show any effect on energy intake or expenditure, but rather, augmented postprandial glycaemia in patients with T2DM receiving long-acting GLP-1 receptor agonists [98]. The mechanism underlying this phenomenon is unclear, but may be related to the stimulation of glucagon by intravenous GIP administration [86,98].

### 3.3. Cholecystokinin (CCK)

CCK is secreted from the enteroendocrine I-cells located in the duodenum and upper jejunum [1]. In mice, there is also evidence that a subset of EECs co-express CCK, GIP, GLP-1 and PYY in the proximal small intestine [83,99]. However, this is unlikely to be the case in humans, since exposure to glucose in the proximal 60 cm segment of the small intestine, while inducing substantial GIP and CCK secretion, does not affect GLP-1 secretion [100]. CCK is secreted within 10–15 min in response to oral ingestion of macronutrients (fat > protein > carbohydrate) [1]. This is critical for subsequent digestion since CCK stimulates the release of digestive enzymes and bile from the pancreas and the gallbladder, respectively.

Exogenous CCK is reported to attenuate the postprandial glycaemic excursion in humans. This effect is secondary to the slowing of gastric emptying, rather than a direct effect on glucose metabolism; intravenous administration of CCK at physiological doses diminishes the glycaemic response to an oral, but not an intraduodenal, glucose load in healthy males [101]. Similarly, in patients with well-controlled T2DM, the administration of CCK at a physiological dose (0.4 pmol/kg/min) was shown to slow gastric emptying and reduce postprandial blood glucose excursions [102]. However, for those with longstanding T2DM, both the secretion and action of CCK appear to be impaired, due probably to the development of autonomic neuropathy [3,103]. In addition to its effects on upper gastrointestinal motor function, CCK has an established role in the regulation of appetite through both vagal and endocrine pathways. In rats, the effect of exogenous CCK to suppress food intake was abolished by small-molecule CCK antagonists or after vagotomy [104]. Similarly, intravenous infusion of CCK at both physiological (0.6–0.8 pmol/kg/min) and supraphysiological doses (1.8 and 2.6 pmol/kg/min) suppresses hunger and energy intake in healthy subjects [105,106,107], effects abolished in the presence of a CCK antagonist [108]. In a population-based study, genetic polymorphisms of the CCK receptor, e.g., increased CCK_H3 haplotype frequency, may be responsible for over-eating in obesity [109]. However, acute administration of exogenous CCK showed a comparable effect on suppressing appetite in non-diabetic obese and lean subjects [110].

### 3.4. Peptide YY (PYY)

PYY is a 36-amino-acid peptide co-released with GLP-1 from L-cells [111]. Like other gastrointestinal hormones, postprandial secretion of PYY is dependent on the composition and load of macronutrients [112,113,114,115]. In contrast to GLP-1 and GIP, enzymatic conversion of PYY1-36 to PYY3-36 by DPP-4 is necessary for the systemic effects of PYY, namely suppression of appetite and slowing of gastric emptying [1].

PYY participates in the regulation of appetite and energy intake. PYY-null mice exhibit increased daily food intake and weight gain when compared with wild type mice, and this phenotype is reversed with PYY3-36 administration [115]. PYY binds to Neuropeptide Y receptor Y2 (NPY2R), which is highly expressed in the hypothalamic arcuate nucleus [116]. That the effect of PYY on energy intake is abolished in NPY2R knockout mice and by the selective NPY2R antagonist BIIE0246, suggests a key role of NPY2R signalling in mediating the effect of PYY to suppress energy intake [117,118]. In healthy humans, postprandial PYY levels are positively correlated with changes in satiety scores [119,120], and intravenous PYY(3-36) infusion (up to 0.8 pmol/kg/min) reduces food intake in a dose-dependent manner [121,122]. Recently, the long-acting PYY3-36 analogue, mAb-cycPYY, was shown to reduce food intake and body weight over 7 days in rhesus macaques, effects further augmented when combined with the GLP-1 receptor agonist, liraglutide [123].

PYY may be also involved in the regulation of postprandial glycaemia, given its effect to slow gastric emptying in both rodents and humans [124,125,126,127]. Furthermore, PYY may influence insulin secretin; PYY1-36, but not PYY3-36, was found to inhibit insulin secretion from the pancreatic β-cells ex vivo [128,129,130], and isolated islets from PYY-knockout mice showed higher glucose-induced insulin levels [131]. PYY-knockout mice exhibit relative hyperinsulinaemia during fasting and postprandially [132]. However, exogenous PYY infusion had little effect on glucose-stimulated insulin in healthy humans [133].

## 4. Regional Differences in Nutrient Absorption and Gastrointestinal Hormone Secretion, and Associated Impact on Postprandial Glycemia and Appetite

### 4.1. Nutrient Absorption

The upper small intestine (duodenum and proximal jejunum) represents a major site of nutrient absorption. Given that the delivery of nutrients into the small intestine is controlled by gastric emptying, it is not surprising that the rate of nutrient absorption (such as glucose) is related directly to the rate of gastric emptying [134]. In the case of glucose, the maximum capacity of absorption of the upper small intestine approximates ~0.5 g/min per 30 cm [135]. Of note, glucose transporters (SGLT-1, GLUT2 and GLUT5) are less abundant in the distal than proximal small intestine [9,136]. Accordingly, intra-ileal administration of glucose is associated with slower absorption than intraduodenal, in both healthy individuals and patients with T2DM [137]. Moreover, duodenal-jejunal bypass improves glucose tolerance, associated with a reduction in SGLT-1-mediated glucose absorption in both obese rats and T2DM patients [138,139,140,141]. Similarly, the expression of the majority of lipid transporters (e.g., FAT/CD36, FATP4 and NPC1L1) decreases from the duodenum and jejunum to the ileum in rodents [142,143,144,145,146], and fatty acid and cholesterol uptake is slower in the distal than proximal small intestine [146,147]. In mice, the ablation of FAT/CD36 and FATP4 is associated with impaired lipid absorption in the proximal [145,148], but not in the distal [145,149] small intestine. However, fatty acid transporters have been shown to be abundantly expressed in both proximal and distal small intestine in humans [150]. The expression of transporters of amino acids and peptides varies substantially along the gastrointestinal tract [16]. While the majority of digestive products of protein are absorbed in the proximal jejunum, a considerable proportion is also absorbed in other small intestinal segments [17]. Amino acid transporters are abundant in the distal jejunum and ileum [151], such that obese patients who have undergone Roux-en-Y gastric bypass (RYGB) exhibit accelerated uptake of amino acids [152]. The absorption of nutrients in the large intestine is minimal. Undigested nutrients are fermented by microbiota localised in the large intestine to produce SCFAs which can be absorbed passively across colonic mucosa.

### 4.2. Gastrointestinal Hormone Secretion

EECs secreting GIP, CCK, GLP-1 and PYY exhibit high regional specificity of distribution along the gastrointestinal tract. Their secretory profiles are, therefore, largely dependent on the region of the gut exposed to intraluminal stimuli. In response to meal ingestion, increments in plasma GIP and CCK usually occur earlier than those of GLP-1 and PYY [153], consistent with the proximal distribution of K- and I-cells, and distal predominance of L-cells. Studies employing intraduodenal infusion of nutrients at different rates that mimic the physiological range of gastric emptying have shown that the secretion of GIP and CCK increases in an approximately linear pattern with increasing rates of infusion in health, obesity and T2DM [154,155,156,157,158,159,160,161]. In obese and T2DM patients, both the GIP and CCK responses to oral meals are increased after Roux-en-Y gastric bypass [162,163], due probably to rapid gastric pouch emptying [164]. However, postprandial GIP secretion is decreased after biliopancreatic diversion, since this procedure bypasses the majority of K-cell rich regions of the small intestine [165,166]. By contrast, intraduodenal infusion of nutrients needs to exceed a threshold (e.g., ~2 kcal/min for glucose) for sufficient nutrient to escape proximal small intestinal absorption and stimulate more distal L-cells; accordingly, the GLP-1 response is minimal to intraduodenal glucose infusion at rates between 1–2 kcal/min but increases substantially in response to infusions of 3–4 kcal/min [155,158,159].

The extent to which nutrients are delivered to the more distal regions of the gut is dependent not only on their rate of entry to the small intestine but also on their digestion and absorption in the upper gut. For example, ablation or inhibition of SGLT-1 that reduces proximal intestinal glucose absorption augments the GLP-1 and PYY responses to oral glucose in rodents [167,168]. In humans, intestinal SGLT-1 inhibition (e.g., by GSK-1614235 [169] or licogliflozin [170]), while reducing GIP secretion, is associated with overall increased, albeit relatively delayed, responses of GLP-1 and PYY to carbohydrate meals. Similarly, malabsorption of carbohydrate induced by an α-glucosidase inhibitor (e.g., acarbose) was shown to increase GLP-1 and PYY secretion in both health and T2DM [171,172]. Alternatively, poorly absorbed carbohydrates, such as tagatose [27], xylose [173] and resistant starch [174], also can induce sustained secretion of GLP-1. Consistent with this principle, consumption of a small amount of tagatose or xylose as a “preload” has been shown to slow gastric emptying and improve the glycaemic response to the subsequent main meal by stimulating GLP-1 secretion in both health and T2DM [27,175]. Treatment with the lipase inhibitor, orlistat, however, has been reported to either increase [176] or decrease [177,178,179] postprandial GLP-1 secretion. These discrepancies may have reflected differences in the test meals (including the forms of dietary fat) and the associated impact of orlistat on their digestion between studies.

Compared with intraduodenal infusion, administration of nutrients directly into the jejunum or ileum is more effective at stimulating GLP-1 and PYY secretion [137,180,181,182,183,184,185,186]. Interestingly, intra-ileal infusion of glucose is also associated with a considerable, albeit relatively lower, GIP response than intraduodenal infusion in both healthy subjects and patients with T2DM [137,183], suggesting that a considerable number of EEC cells capable of secreting GIP are found even in the distal small intestine. The large intestine represents another major source of GLP-1 and PYY production. Microbial metabolites, including SCFAs and secondary bile acids, are known to stimulate GLP-1 and PYY secretion [47,187,188,189,190,191,192,193] and may also induce differentiation of stem cells towards L-cells [194]. Inhibition of ileal ASBT (by elobixibat), increasing the exposure of the large intestine to bile acids, is therefore associated with an increase in GLP-1 and PYY secretion in humans [195]. In both healthy individuals and patients with T2DM [196,197], rectal administration of primary bile acid, taurocholic acid (TCA), has also been shown to stimulate GLP-1 and PYY secretion in a dose-dependent manner, although the PYY response seems to be more robust than that of GLP-1.

### 4.3. Regulation of Postprandial Glycaemia and Appetite

As discussed, the upper gut coordinates the delivery of nutrients for intestinal digestion and absorption, and is primarily responsible for the release of GIP and CCK after meals, whereas the interaction of intraluminal nutrients and bioactive compounds with the distal gut gives rise to the secretion of both GLP-1 and PYY. These variations in nutrient absorption and gastrointestinal hormone secretion along the gastrointestinal tract are of major relevance to the regulation of postprandial glycaemia and appetite.

It is now widely recognised that the rate of gastric emptying represents a major determinant of the glycaemic profile in response to carbohydrates in both health and diabetes [198,199]. While obesity per se does not seem to have a major impact on gastric emptying [200], recent evidence suggests that gastric emptying in patients with “early-stage” uncomplicated type 1 and 2 diabetes is more rapid than in non-diabetic controls [199,201], which may predispose them to glucose intolerance. By contrast, in patients with longstanding diabetes who have poor glycaemic control and autonomic dysfunction, gastric emptying is often abnormally delayed [202]. Nevertheless, nutritional and/or pharmacological strategies that slow gastric emptying have been shown to attenuate postprandial glycaemic excursions in both type 1 and 2 diabetes [57,203,204,205]. However, it should be noted that the relationship between the rise in postprandial blood glucose and the gastric emptying rate is not necessarily linear. Intraduodenal glucose infusion at 2 kcal/min results in a much greater increase in blood glucose levels than 1 kcal/min in healthy humans, while minimal additional increase occurs in glycaemia at rates of 3 or 4 kcal/min [155,158,159], due probably to the increasing contribution of the distal gut to provide counter-regulation to glycaemic excursions.

In health, GIP contributes to approximately 50% of the incretin effect [206] and may serve to stabilise blood glucose by stimulating glucagon secretion during hypoglycaemia [87]. However, the loss of the insulinotropic effect of GIP in T2DM, and the potential for GIP to increase fat deposition, have rendered it an unappealing target for the management of T2DM. Recently, novel compounds with dual GIP and GLP-1 receptor agonism have been developed to treat T2DM, with promising glucose-lowering efficacy [96]. However, as mentioned earlier, the relative contribution of GIP receptor agonism to the overall metabolic benefits of these compounds remains unclear. The rapid release of CCK in response to meal ingestion is necessary for the digestion of complex macronutrients, particularly fat, so it represents a determinant of subsequent gastric emptying and appetite responses.

**Figure 3:**
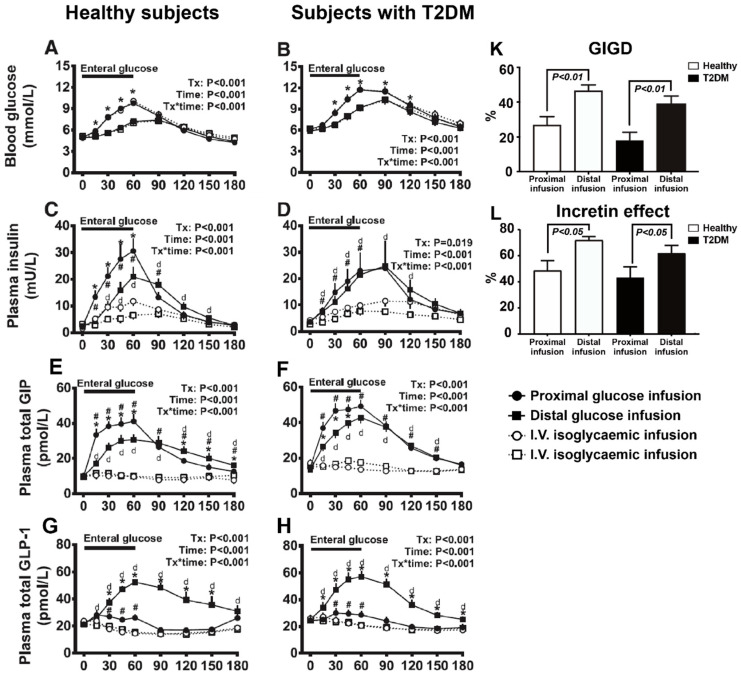
Comparative effects of proximal and distal small intestinal glucose exposure on glycaemia, incretin hormone secretion, and the incretin effect in both healthy individuals and patients with type 2 diabetes (T2DM) (*n* = 10 each). (**A**,**B**) Blood glucose levels, (**C**,**D**) plasma insulin, (**E**,**F**) plasma total glucose-dependent insulinotropic polypeptide (GIP), (**G**,**H**) plasma total glucagon-like peptide 1 (GLP-1), (**K**) Gastrointestinal-mediated glucose disposal (GIGD), and (**L**) Incretin effect. * *p* < 0.05 for proximal vs. distal enteral glucose infusion; # *p* < 0.05 for proximal enteral vs. corresponding i.v. glycaemic glucose infusion; d*p* < 0.05 for distal enteral vs. corresponding i.v. glycaemic glucose infusion. Data are mean ± SEM. (Figures are adapted from reference [137], with permission from Diabetes Care, 2019).

However, when nutrients are administered intraduodenally, a threshold of the caloric load is required to achieve suppression of appetite [207], indicative of a greater relevance of stimulating the distal gut to the control of appetite. Indeed, relative to the upper gut, the lower gut appears to be more effective at mediating postprandial glucose metabolism and suppressing appetite due to substantially augmented GLP-1 and PYY secretion. Recently, the comparative effects of the proximal and distal small intestine on postprandial glucose metabolism were evaluated using targeted intraluminal glucose infusion in both healthy individuals and patients with T2DM [137]. In both groups, intra-ileal administration of glucose (2 kcal/min over 60 min) was associated with substantially greater GLP-1 secretion, incretin effect and gastrointestinal-mediated glucose disposal (GIGD), when compared with intraduodenal infusion (Figure 3). That the absorption of glucose occurs at a lower rate in the ileum, probably because of fewer glucose transporters in the distal gut, may not only attenuate the glycaemic response to glucose infusion but also allow EECs to be stimulated over a longer duration than those in the proximal gut [137]. In a similar study setting, Poppitt and colleagues compared the effects of a small load of glucose (~0.65 kcal/min over 90 min), administered into either the ileum or the duodenum, on gastrointestinal hormone secretion, appetite and food intake in healthy subjects. In this study, ileal infusion of glucose-induced greater GLP-1 and PYY secretion and less food intake than did intraduodenal infusion [183]. Compared with oral ingestion or duodenal perfusion, delivering fat or protein into the ileum also induces a greater suppression of food intake in healthy humans [185,186,208]. Moreover, administration of a relatively small load of lauric acid (5 g; 20 kcal) in enterically coated pellets for release in the ileum and colon has been shown to stimulate sufficient GLP-1 secretion to improve the glycaemic response to a standardised breakfast and lunch in patients with T2DM [209]. Similarly, the ileocolonic delivery of mixed bile acids (1 g/day) increases GLP-1 secretion and reduces postprandial blood glucose levels in patients with obesity and T2DM during a 4-week treatment [210]. Alternatively, EEC stimuli can be delivered through rectal administration; rectal perfusion with TCA has been shown to stimulate GLP-1 and PYY secretion, and suppress appetite scores in health [196] and reduces energy intake and glycaemia in T2DM [197]. Accordingly, enhancing the exposure of the distal gut to nutrients, and associated bioactive compounds such as bile acids, either by pharmacological inhibition of nutrient digestion and absorption in the upper gut [171,172], surgical reconstruction of the gastrointestinal tract (such as Roux-en-Y gastric bypass) [211,212], or endoscopic implantation of a duodenal-jejunal bypass sleeve device [140,141], has been shown to improve blood glucose control in T2DM and reduce body weight in obesity. The causal links of these metabolic outcomes to GLP-1 and PYY signalling have been further validated in T2DM patients undergoing Roux-en-Y gastric bypass, in whom glucose tolerance is attenuated by the GLP-1 receptor antagonist exendin9-39) [213,214], while energy intake is increased with either GLP-1 receptor antagonism, or inhibition of PYY activation using a DPP-4 inhibitor [215].

In recognition that the gut microbiota are an essential regulator of the host energy metabolism [216], and that insulin resistance, obesity and T2DM are linked to dysbiosis [217], the role of the large intestinal bacteria in the regulation of glycaemia and food intake is now receiving increasing attention. While the mechanisms by which the gut microflora participate in the regulation of metabolic homeostasis remain incompletely understood, many of their metabolites are linked to gastrointestinal hormone secretion [218,219]. For example, SCFAs, including acetate, butyrate and propionate, have been shown to stimulate GLP-1 and PYY from colonic L-cells in a dose-dependent manner [187,188,192] and to enhance insulin secretion either directly or indirectly in both rodents and humans [187,188,189,190,191,192]. Oral supplementation with propionate and butyrate improves blood glucose control and promotes weight loss in rats [220]. In obese individuals, acute administration of inulin-propionate ester (10 g), designed to be released in the colon, was shown to increase postprandial GLP-1 and PYY concentrations and decrease energy intake, without affecting gastric emptying [191]. Moreover, the administration of inulin-propionate ester (10 g/day) over 24 weeks showed a tendency to reduce body weight in obese subjects [191]. However, this phenomenon is complicated by evidence of a central effect contributing to the suppression of energy intake after a single dose of colonic inulin-propionate ester, independent of changes in peripheral GLP-1 or PYY concentrations [221].

## 5. Summary

The gastrointestinal tract serves not only as the site of nutrient digestion and absorption but also as an endocrine organ secreting a variety of gastrointestinal hormones as a result of the complex interaction between ingested nutrients, bioactive compounds and EECs to regulate postprandial glucose metabolism and energy intake. Given the major difference in the distribution of EECs between the upper and lower gut, the load and delivery of nutrients, as well as the digestive processes in the gastrointestinal tract, have major implications on how these EECs are stimulated. Exposure of the upper gut to nutrients is associated with predominantly GIP and CCK release, whereas increasing the delivery of nutrients to the distal small intestine and colon is associated with augmented secretion of GLP-1 and PPY. These distal gut hormones appear more potent in mediating postprandial glucose metabolism and suppressing energy intake than those secreted from the proximal gut. Accordingly, the distal gut is becoming an appealing target for the management of T2DM and obesity, using nutritional, pharmacological or surgical approaches to increase its exposure to nutrients and other bioactive compounds. Future development in this area is likely to yield novel therapies for T2DM and obesity of high efficacy without the need for surgical procedures.
